# Soluble Biomarkers of Cartilage and Bone Metabolism in Early Proof of Concept Trials in Psoriatic Arthritis: Effects of Adalimumab Versus Placebo

**DOI:** 10.1371/journal.pone.0012556

**Published:** 2010-09-03

**Authors:** Arno W. R. van Kuijk, Jeroen DeGroot, Rishma C. Koeman, Nico Sakkee, Dominique L. Baeten, Danielle M. Gerlag, Paul P. Tak

**Affiliations:** 1 Division of Clinical Immunology and Rheumatology, F4-218, Academic Medical Center/University of Amsterdam, Amsterdam, The Netherlands; 2 Business Unit Biosciences, The Netherlands Organization for Applied Scientific Research (TNO) Quality of Life, Leiden, The Netherlands; Hôpital Cochin, France

## Abstract

**Background:**

There is growing interest in soluble biomarkers that could be used on the group level for screening purposes in small proof of principle studies during early drug development. We investigated early changes in serum levels of several candidate biomarkers involved in cartilage and bone metabolism following the initiation of adalimumab as a prototypic active treatment in psoriatic arthritis (PsA) compared to placebo.

**Materials and Methods:**

Twenty-four PsA patients were randomized to receive either adalimumab 40 mg s.c. every other week or placebo for 4 weeks, followed by an open label extension phase. Serum samples were obtained at baseline and after 4 and 12 weeks of treatment and analyzed for levels of CPII and PINP (synthesis of type II and type I procollagen), melanoma inhibitory activity (MIA) (chondrocyte anabolism), matrix metalloproteinase (MMP)-3, C2C and cartilage oligomeric matrix protein (COMP) (type II collagen degradation), osteocalcin (OC) (bone formation), NTX-I and ICTP (both type I collagen degradation).

**Results:**

After 4 weeks, there was a significant decrease in serum MMP-3 levels in adalimumab-treated patients (P<0.005), while no change was observed in the placebo group. A significant increase in serum MIA was noted after adalimumab therapy (P<0.005) but not after placebo treatment. After 12 weeks, there was a marked reduction in serum MMP-3 in both groups (P<0.005), whereas other markers did not show significant changes compared to baseline.

**Conclusion:**

MMP-3 and MIA could serve as soluble biomarkers associated with inflammation as well as joint remodelling and destruction and may, together with clinical evaluation and in combination with other biomarkers, assist in distinguishing between effective and ineffective therapy in small, proof-of-principle studies of short duration in PsA.

**Trial Registration:**

Current Controlled Trials ISRCTN23328456

## Introduction

The peripheral arthritis in psoriatic arthritis (PsA) is characterized by progressive destruction in the majority of patients [1]. The articular damage develops over months to years, but can often be detected within 2 years of the first consultation of a rheumatologist [2]. The chronic inflammation of the synovial membrane is thought to be responsible for degradation of cartilage and bone, in part by locally produced cytokines and proteinases. Treatment with conventional antirheumatic drugs can improve the clinical manifestations and inhibit permanent joint damage, but these drugs are not effective in or tolerated by all patients. New targeted therapies, such as tumor necrosis factor (TNF) blockade, have expanded therapeutic possibilities, but not all patients respond well [3], [4]. Therefore, there is still a need for new and better treatment options, and several new therapeutic strategies are in the pipeline of pharmaceutical industry. The increase in the development of numerous new, targeted therapies clearly raises the need for sensitive biomarkers which could be used on the group level for early selection of potentially effective treatments.

It has recently been proposed to use small, intensive studies providing a high density of data to obtain initial proof of principle in an early stage of drug development before larger, conventional clinical trials are conducted to determine whether the effects of a new treatment are clinically meaningful [5]–[7]. In this design additional biological data is collected alongside conventional measures of disease activity to determine if there is a biological effect related to the mechanism of action of the drug tested. We have previously shown that synovial biomarkers may be used in this context in clinical trials in both rheumatoid arthritis (RA) and PsA [6], [7]. The use of soluble biomarkers could increase the feasibility of this approach, as synovial biopsy is not routinely available in all centers. Moreover, various biomarkers could be combined together with clinical evaluation for initial assessment of efficacy.

The treatment objectives in PsA are not limited to improvement of clinical signs and symptoms, but include protection against joint destruction. Since structural damage outcomes require lengthy clinical trials, the availability of biomarkers associated with these long-term endpoints would be an attractive therapeutic development strategy, especially in early phase trials when decisions are being made whether and how to proceed with pivotal clinical trials. For this reason, biologic markers of bone and cartilage metabolism are of particular interest, because they may reflect changes related to the integrity of the affected joints [8], [9].

Bone matrix is mainly composed of type I collagen, while type II collagen is the main collagen in articular cartilage. Type I collagen telopeptide fragments, such as C-terminal cross-linked telopeptide of type I collagen (CTX-I and ICTP) and N-terminal cross-linked telopeptide of type I collagen (NTX) in serum are currently considered to be sensitive markers of bone resorption [10]. ICTP is released from bone type I collagen by activity of matrix metalloproteinases (MMPs), while CTX-I is generated by cathepsin K activity, but not MMPs [11]. Pro-collagen serum type I N-terminal propeptide (PINP) and osteocalcine (OC) are markers of bone formation.

Type II collagen synthesis can be detected by measurement of C-propeptide of type II collagen (CPII), while cartilage degradation by collagenases can be detected in serum by cleavage products of type II collagen, such as Col2-3/4C (C2C) and cartilage oligomeric matrix protein (COMP) [12]. Of importance, several studies in patients with inflammatory arthritis have shown that some of these markers of bone and cartilage metabolism are associated with progression of radiographic joint damage [13]–[15], and that their tissue expression and serum levels may change after initiation of effective (biologic) therapy [13], [16]–[19]. Similarly, it has been suggested that melanoma inhibitory activity (MIA), also known as cartilage-derived retinoic acid-sensitive protein (CD-RAP), a marker for chondrocyte anabolism, could be used as a biomarker in patients with RA [20]. MIA is suppressed by pro-inflammatory cytokines in vitro, and serum MIA levels are increased after one year of infliximab treatment in patients with inflammatory arthritis [21].

The early effects of active treatment on synovial biomarkers, following the initiation of adalimumab as a prototypic active treatment compared to placebo, have recently been reported for this study cohort of patients with active PsA [6]. As a secondary endpoint in this study, we investigated early changes in serum levels of several candidate soluble biomarkers involved in formation and degradation of cartilage and bone, following the initiation of adalimumab treatment compared to placebo in the same cohort of patients. The results on soluble biomarkers presented in this paper are supplementary to the results previously published on synovial biomarkers. The ultimate goal of our studies is to identify biomarkers that could be used in combination as a screening tool to differentiate on the group level between potentially effective and ineffective treatments in small proof of principle studies during an early stage of clinical development.

## Materials and Methods

### Study protocol

The study protocol was approved by the Medical Ethical Committee of the Academic Medical Centre/University of Amsterdam (ref: MEC 05/162, ISRCTN23328456), and all patients gave their written informed consent. The protocol for this trial and supporting CONSORT checklist are available as supporting information; see Checklist S1 and Protocol S1.

### Patients

Twenty-four active PsA patients fulfilling the CASPAR classification criteria for PsA [22], were randomized to receive adalimumab 40 mg s.c. every other week (n = 12) or matched placebo (n = 12) for 4 weeks, followed by an open label extension phase during which all patients were treated with adalimumab. Most patients (n = 16) had polyarticular involvement according to the Moll and Wright classification [23], a minority had an oligoarticular phenotype (n = 7) or predominantly distal interphalangeal involvement (n = 1). Two of the patients with polyarticular disease also had axial involvement. The baseline demographic data are presented in Table S1. The clinical results of this study have been reported earlier [6]. Serum samples and clinical assessments were obtained at baseline and after 4 and 12 weeks of treatment. In addition, erythrocyte sedimentation rate (ESR) and C-reactive protein (CRP) were determined. The disease activity score evaluated in 28 joints (DAS28), which has been shown to discriminate between active drug and placebo in clinical trials in PsA, was chosen to monitor changes in clinical disease activity after therapy [24]; [25]

### Measurement of markers of cartilage and bone metabolism

N-MID Osteocalcin (ELISA, Nordic Bioscience Diagnostics A/S, Herlev, Denmark), PINP (UniQ RIA, Orion Diagnostica, Espoo, Finland), NTX (ELISA, Osteomark, Inverness Medical Professional Diagnostics, Princeton, New Jersey, USA), ICTP (UniQ EIA, Orion Diagnostica, Espoo, Finland), CPII (ELISA, IBEX Pharmaceuticals Inc, Montreal, Canada), C2C (ELISA, IBEX Pharmaceuticals Inc, Montreal, Canada), COMP (ELISA, AnaMar Medical, Goteborg, Sweden), and serum MMP-3 (Quantikine ELISA, R&D Systems, Abingdon, UK) levels were were determined at TNO (Leiden, The Netherlands) according to the instruction provided by the assay's manufacturers. Also, serum MMP-3 was measured. MIA, a marker for cartilage anabolism, was determined using a commercially available one step ELISA kit (Roche Diagnostics, Mannheim, Germany) following the manufacturer's instruction.

*Statistical analysis* Parameters with a normal distribution were analyzed using a paired samples t-test, while parameters with a skewed distribution were assessed with the Wilcoxon signed rank test. In addition, each of the endpoints at week 4 (including ESR and CRP) was analyzed using a repeated measure analysis of covariance model (ANCOVA). The model included terms for treatment as a fixed effect and the baseline measurement as a covariate with the aim to assess the treatment difference. Correlations of changes in clinical parameters and serum markers were analyzed with Spearman's rank correlation.

## Results

### Clinical response

We have previously described the primary clinical outcome after 4 weeks adalimumab versus placebo treatment, as well as the results of synovial tissue analysis [6]. At week 12, when all 24 patients were treated with adalimumab, the mean DAS28 in all patients decreased from 4.86±1.14 at baseline to 2.84±1.36 at week 12 (P<0.001), mean CRP was reduced from 15.0±19.5 to 2.8±4.9 mg/l (P = 0.003) and ESR decreased from 23.3±19.8 to 7.2±6.1 mm in 1^st^ hour (P<0.001).

### Markers of type I collagen metabolism (bone) and type II collagen metabolism (cartilage)

The results of the markers tested are presented in Table S2. After 4 weeks of adalimumab therapy there was a significant decrease in median (± SD) serum MMP-3 levels in adalimumab treated patients from 41.0±35.1 to 14.5±12.6 ng/ml (P<0.005), while no change was observed in the placebo group. After 12 weeks, when all patients were treated with adalimumab, median serum (± SD) MMP-3 levels in both groups were reduced significantly (P<0.005) as shown in Table S2 and Figure S1.

Median (± SD) serum MIA levels increased significantly after treatment with adalimumab from 5.77±3.3 at baseline to 6.74±4.3 ng/ml at week 4 (P<0.005), while serum levels after placebo treatment were unchanged. After 12 weeks the change in MIA did not reach statistical significance in either group, as shown in Table S2 and Figure S2.

Overall, no significant early change in the serum levels of these bone and cartilage markers was observed. There was a trend towards a reduction of median (± SD) serum level NTx in the adalimumab group from 91.9±34.3 at baseline to 75.3±23.8 nM BCE after 4 weeks of treatment (P = 0.078), while NTx levels in the placebo group remained unchanged. There were no significant changes in NTx at week 12. We also found a trend towards an increase of median (± SD) CPII concentrations in the adalimumab treated group from 668±169 at baseline to 765±167 ng/ml after 4 weeks (P = 0.053), while CPII levels in the placebo group did not change. At week 12 there was a non-significant reduction of CPII level in both groups. The biological meaning of these findings is at present uncertain.

When the repeated measure ANCOVA was applied for each of the endpoints at week 4, the effect of active treatment was significant for the reduction of ESR (P = 0.001), CRP (P = 0.01), and serum MMP-3 (P = 0.006), as well as for the increase of serum MIA level (P = 0.013), indicating that these biomarkers could distinguish between effective and ineffective treatment (Table S3).

### Correlation between clinical improvement and changes in serum biomarkers

Change in DAS28 at week 4 was strongly correlated with change in CRP (rho 0.755, P<0.001), ESR (Spearman's rho 0.737, P<0.001), MMP-3 (rho 0.709, P<0.01) and MIA (rho -0.507, P<0.01) as shown in Table S3.

## Discussion

This placebo-controlled trial with adalimumab in patients with PsA was conducted to explore if serum biomarkers of cartilage and bone metabolism could be used to screen for potential efficacy on the group level in small, early phase trials of short duration. The recent rise in new drugs discovered may have consequences for the way these potential novel therapies are tested in patients. This is usually done in relatively large, placebo-controlled clinical trials. It has become increasingly difficult to enrol a large number of patients with active disease in these trials, because of the growing number of compounds to be tested, and the fact that effective treatment is available for many patients. Therefore, in an early stage of drug development it could be favourable to test potential drugs in a short intensive proof of principle trial for selection purposes, using a small number of patients in which a large amount of data is collected. If no clinical or biological effect is found in such a trial, the compound appears less likely to be effective in a larger clinical trial of longer duration. If, on the other hand, there is a clear clinical and/or biological effect, the compound may be effective, and could be considered for conventional phase 2 clinical trials to determine whether these effects appear clinically meaningful. Our results suggest that MMP-3 and perhaps MIA may be instrumental as a screening method to test new drug candidates for PsA requiring relatively small numbers of subjects. Obviously, the results presented here need to be confirmed and validated in a comparable study design using another effective drug with a different mechanism of action, for instance ustekinumab [26], for which this study provides the rationale.

We found a rapid and strong reduction of serum MMP-3, a matrix metalloproteinase that is involved in inflammation as well as degradation of cartilage and bone, after initiation of adalimumab therapy. The effect of treatment on reduction of MMP-3 in serum was comparable with the reduction of measures of inflammation (CRP levels and ESR) in the ANCOVA model. The change in serum MMP-3 levels also strongly correlated with clinical improvement as measured by DAS28. The observed changes in MMP-3 concentration are interesting for 2 reasons. First, this may reflect a change in cartilage degradation in the affected joints, while the assessment of CRP and ESR merely represents a change in inflammation. Secondly, it is of particular interest in patients with PsA, because these patients do not always have elevated levels of CRP and ESR, even while their disease is active. The decrease in MMP-3 was sustained up to 12 weeks.

It has been shown that serum MMP-3 levels are elevated in patients with inflammatory arthritis compared with healthy controls [27]–[30]. There is still controversy, however, whether baseline MMP-3 level or change in MMP-3 level after treatment are good predictors for future radiographic progression in individual patients. While some authors find an association between baseline MMP-3 and subsequent radiographic joint damage [28], [31]–[33], some do not [34], [35]. Evidence for a correlation between radiographic progression and reduction of MMP-3 level after treatment was reported by only one group [36]. The rapid decrease of serum MMP-3 level after TNF blocking therapy is in line with earlier observations in patients with spondyloarthritis (SpA). In a study with 22 patients with active SpA who were treated with infliximab (n = 12 including 6 PsA patients) or placebo (n = 10) [19], there was a significant reduction of serum MMP-3 level one week after start of infliximab treatment, which was sustained up to week 12, while no changes were observed in the placebo treated patients. Similarly, an open-label study showed a significant decrease in serum MMP-3 levels 12 weeks after initiation of etanercept treatment in 20 SpA patients (including 6 PsA patients) [37]. In patients with ankylosing spondylitis treated with adalimumab (n = 38), serum MMP-3 concentration significantly decreased after 12 weeks of treatment, while it was unchanged after placebo [38].

We also found an increase of serum MIA, a marker for chondrocyte activity, after 4 weeks of adalimumab treatment. The change in serum MIA levels correlated with clinical improvement as measured by DAS28 at week 4 in the adalimumab treated patients. It has been demonstrated that serum MIA levels are reduced in patients with active inflammatory arthritis when compared to healthy controls [20], [21]. A 39% increase of serum MIA concentration was observed in 15 RA patients one year after start with infliximab, as compared to a 20% increase in 25 SpA patients [21]. In a subset of 7 RA patients receiving infliximab, it was demonstrated that this increase in serum MIA level was detectable from week 30 and beyond. Of interest, we observed a swift increase in serum MIA concentration after initiation of adalimumab treatment in PsA patients after 4 weeks, but after 12 weeks no significant effect could be detected. This could indicate that initiation of adalimumab therapy in PsA patients induces a short period of enhanced chondrocyte activity in PsA patients after initiation of adalimumab therapy, which returns to normal after several weeks. However, the control group did not show an increase in MIA level after initiation of adalimumab. More data are obviously needed to understand the underlying mechanism of MIA regulation and the effect of TNF blockade.

Only small (trends towards) changes were observed in the other markers tested after initiation of TNF blocking therapy. Although these trends may reflect an important biological mechanism, these markers may not be the best candidates to assess effective therapy in early phase clinical trials of short duration. MMP-3 seems to be more responsive to change than the other markers involved in cartilage breakdown. The lack of change of the other markers might be explained in part by the relatively short study duration. The goal of our study was to identify biomarkers that may help to distinguish between effective and ineffective therapy in proof of principle studies of short duration. We cannot exclude the possibility that changes would be seen for CPII and COMP after more prolonged treatment

In conclusion, adalimumab therapy in PsA is associated with a specific and highly significant decrease in serum MMP-3 and an increase in serum MIA levels, suggesting that the measurement of these soluble biomarkers, known to be associated with the effects on the structural integrity of the joints, could be useful for screening purposes on the group level in proof of principle trials during early drug development.

## Supporting Information

Checklist S1 Consort checklist.
(0.19 MB DOC)

Figure S1 Changes in serum levels of MMP-3 in relationship to treatment. Median and interquartile ranges are shown for serum MMP-3 concentrations in ng/ml at baseline and weeks 4 and 12 for the patients originally randomized to receive adalimumab (panel A), or placebo (panel B). After 4 weeks of adalimumab therapy, there was a significant decrease in median (± SD) serum MMP-3 concentration in adalimumab-treated patients from 41.0±35.1 to 14.5±12.6 ng/ml (* P<0.005), and this reduction was sustained at week 12 (panel A). No change in median serum MMP-3 concentration was observed in the placebo group at week 4, but after open label adalimumab treatment from week 4 to week 12, there was a decrease in MMP-3 levels in this group as well (* P<0.005, panel B).
(0.07 MB TIF)

Figure S2 Changes in serum levels of MIA in relationship to treatment. Median and interquartile ranges are shown for serum MIA concentrations in ng/ml at baseline and weeks 4 and 12 for the patients originally randomized to receive adalimumab (panel A), or placebo (panel B). After 4 weeks, median(± SD) serum MIA concentration in adalimumab-treated patients increased significantly from 5.77±3.3 at baseline to 6.74±4.3 at week 4 (* P<0.005), but this was not significant at week 12 (panel A). No significant changes were noted in serum MIA concentration in the placebo-treated patients at week 4, or at week 12 after receiving open label adalimumab from week 4 to 12 (panel B).
(0.06 MB TIF)

Protocol S1 Trial protocol.
(0.50 MB DOC)

Table S1 Demographic and clinical features of the 24 patients with psoriatic arthritis (PsA) enrolled in the study. All values are presented as mean (range) except where indicated otherwise. PA, polyarticular; OA, oligoarticular; DIP, predominant distal interphalangeal; RF, rheumatoid factor; ACPA, anti-citrillunated protein antibody; MTX, methotrexate; ESR, erythrocyte sedimentation rate; CRP, C-reactive protein; DAS28, disease activity score in 28 joints; VAS, visual analogue scale; PASI, psoriasis area end severity index.
(0.04 MB RTF)

Table S2 Results of markers of collagen type I and collagen type II in placebo and adalimumab treated groups. All values are presented as median (standard deviation, SD). ** P<0.005. NTx, N-terminal telopeptide of type I collagen; PINP, pro-collagen type I N-terminal propeptide; ICTP, C-terminal telopeptide of type I collagen; OC, osteocalcine; MMP-3, matrix metalloproteinase-3; MIA, melanoma inhibitory activity; CPII, C-propeptide of type II collagen; COMP, cartilage oligomeric matrix protein; C2C, Col2-3/4C.
(0.04 MB DOC)

Table S3 P-values of the repeated measure ANCOVA for each marker, including ESR and CRP. The correlation between each marker, including ESR and CRP, and change in the disease activity evaluated in 28 joints (DAS28) are presented as Spearman rho (P-value).
(0.03 MB DOC)
