# Supplementary figures and images for: Soluble Biomarkers of Cartilage and Bone Metabolism in Early Proof of Concept Trials in Psoriatic Arthritis: Effects of Adalimumab Versus Placebo
Source: PLoS One. 2010 Sep 3;5(9):e12556. doi: 10.1371/journal.pone.0012556 (PMC2937309; doi:10.1371/journal.pone.0012556)

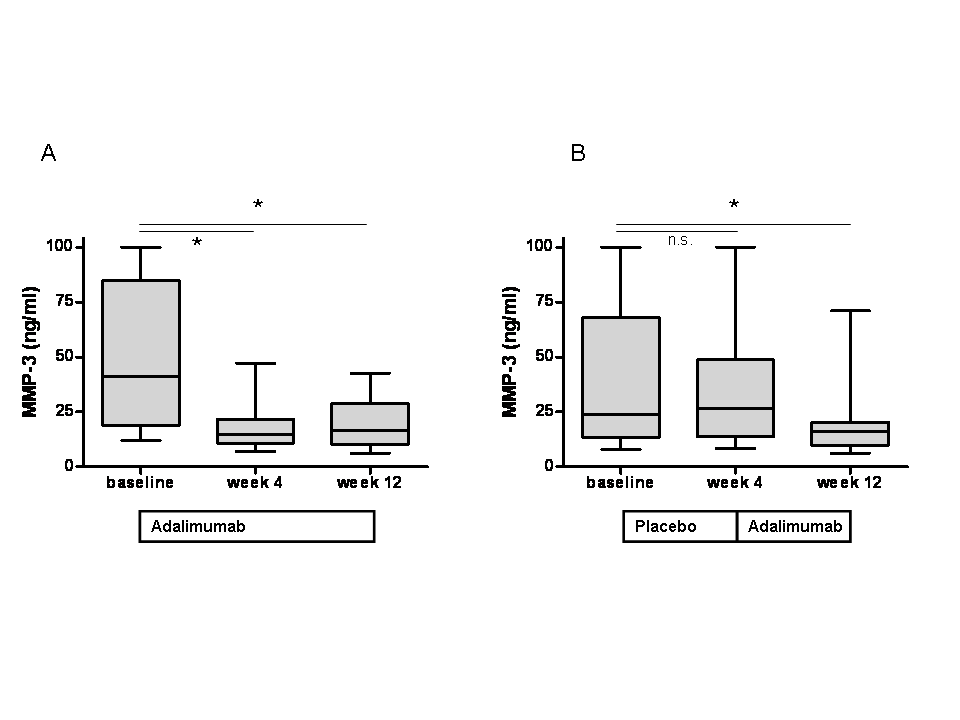

Supplement: Figure S1 — Changes in serum levels of MMP-3 in relationship to treatment. Median and interquartile ranges are shown for serum MMP-3 concentrations in ng/ml at baseline and weeks 4 and 12 for the patients originally randomized to receive adalimumab (panel A), or placebo (panel B). After 4 weeks of adalimumab therapy, there was a significant decrease in median (± SD) serum MMP-3 concentration in adalimumab-treated patients from 41.0±35.1 to 14.5±12.6 ng/ml (* P<0.005), and this reduction was sustained at week 12 (panel A). No change in median serum MMP-3 concentration was observed in the placebo group at week 4, but after open label adalimumab treatment from week 4 to week 12, there was a decrease in MMP-3 levels in this group as well (* P<0.005, panel B). (0.07 MB TIF) [file pone.0012556.s002.tif]

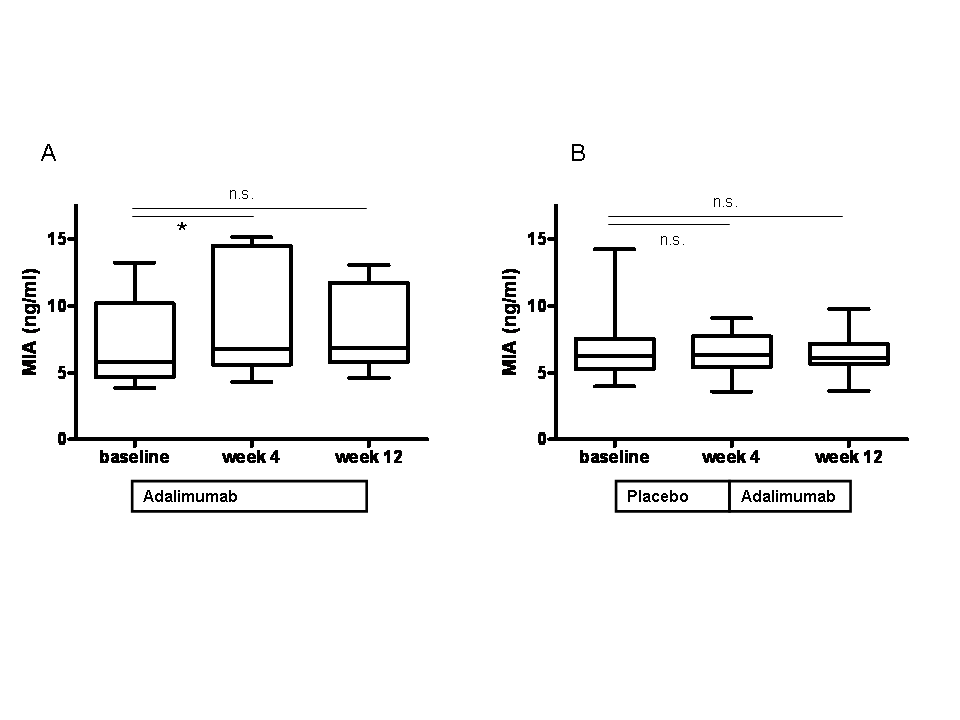

Supplement: Figure S2 — Changes in serum levels of MIA in relationship to treatment. Median and interquartile ranges are shown for serum MIA concentrations in ng/ml at baseline and weeks 4 and 12 for the patients originally randomized to receive adalimumab (panel A), or placebo (panel B). After 4 weeks, median(± SD) serum MIA concentration in adalimumab-treated patients increased significantly from 5.77±3.3 at baseline to 6.74±4.3 at week 4 (* P<0.005), but this was not significant at week 12 (panel A). No significant changes were noted in serum MIA concentration in the placebo-treated patients at week 4, or at week 12 after receiving open label adalimumab from week 4 to 12 (panel B). (0.06 MB TIF) [file pone.0012556.s003.tif]
