# Supplementary material for: Soluble Biomarkers of Cartilage and Bone Metabolism in Early Proof of Concept Trials in Psoriatic Arthritis: Effects of Adalimumab Versus Placebo
Source: PLoS One. 2010 Sep 3;5(9):e12556. doi: 10.1371/journal.pone.0012556 (PMC2937309; doi:10.1371/journal.pone.0012556)
Supplement: Protocol S1 — Trial protocol. (0.50 MB DOC) [file pone.0012556.s004.doc]

# version 16 may 2006

# Effects of adalimumab (Humira®) on biomarkers in skin and synovial tissue in patients with psoriatic arthritis

# Arno W.R. van Kuijk, M.D.^1^

# Gamze Piskin, M.D., Ph.D.^2^

# Tom J.M. Smeets, Ph.D. ^1^

# Danielle M. Gerlag, M.D. ^1^

# Marcel B. M.Teunissen, Ph.D.^2^

# Menno A. de Rie, M.D., Ph.D. ^2^

# Jan D Bos, M.D., Ph. D. ^2^

# Paul P. Tak, M.D., Ph.D. ^1^

# ^1^Division of Clinical Immunology and Rheumatology, AMC

# ^2^Department of Dermatology, AMC

# Corresponding address:

# Prof. Dr. P.P. Tak

# Division of Clinical Immunology and Rheumatology

# Academic Medical Center/University of Amsterdam, F4-218

# PO Box 22700

# 1100 DE Amsterdam

# Tel: + 31 20 5662171

# Fax: + 31 20 6919658

# P.P.Tak@amc.uva.nl

**study synopsis**

| **TITLE** | | Effects of adalimumab (Humira®) on biomarkers in skin and synovial tissue in patients with psoriatic arthritis |
| --- | --- | --- |
| **STUDY SITE** | | Division of Clinical Immunology and Rheumatology  Department of Dermatology  Academic Medical Center, Amsterdam, The Netherlands |
| PHASE | | 4 |
| **INDICATION** | | Psoriatic arthritis |
| **STUDY OBJECTIVES** | | The primary objective is to study changes in synovial and skin inflammation and cytokine expression in serial biopsy samples following administration of adalimumab therapy to subjects with active psoriatic arthritis.  The secondary objectives of this study are to (i) assess clinical response (ii) compare immunohistochemical analysis and Polymerase Chain Reaction (PCR) analysis of cytokines in tissue samples (iii) to assess changes in gene expression in synovial and skin tissue by microarray analysis (iv) to assess changes in serum biomarkers |
| **MEDICATION** | Adalimumab or placebo for 4 weeks followed by an open label extension study for in total 12 weeks. | |
| **DESIGN** | | Single centre, double-blind, randomized, placebo-controlled study in subjects with clinically active psoriatic arthritis who have have persistent disease activity during treatment with methotrexate (MTX) and who are on stable MTX treatment. Synovial and skin biopsies will be obtained before and after 4 weeks of treatment with adalimumab subcutaneously. |
| **POPULATION** | | Males and females with a diagnosis of psoriatic arthritis |
| **SAMPLE SIZE** | | 24 patients |
| **SAFETY VARIABLES** | | Physical examination, vital signs, adverse events, standard hematology and clinical chemistry laboratory, urinalysis. Chest X-ray, PPD and β-HCG pregnancy test (for women) will be performed at screening |
| EFFICACY VARIABLES | | Immunohistologic studies of synovium from an affected joint; immunohistologic studies on skin biopsies of lesional skin; exploratory studies using Q-PCR and microarray analysis; psoriasis area and severity index; body surface area percentage; tender joint score and swollen joint score (based on 68 joints); patient pain score VAS; patient disease activity VAS; physician global assessment of PsA disease activity; CRP, ESR |

| **schedule of assessments** | | | | |
| --- | --- | --- | --- | --- |
|  | screening (-2 weeks) | baseline (week 0) | week 4 | week 12 |
| medical history | X |  |  |  |
| physical examination | X |  |  | X |
| vital signs | X |  |  |  |
| Hematology | X | X | X | X |
| Chemistry | X | X | X | X |
| urinalysis | X | X | X | X |
| chest X ray | X |  |  |  |
| PPD skin test | X |  |  |  |
| serologic test (HIV, HBV, HCV) | X |  |  |  |
| rheumatoid factor | X |  |  |  |
| Anti-CCP antibodies | X |  |  |  |
| Serum for future proteomics |  | X | X | X |
| serum b-hcg | X |  |  |  |
| joint score | X | X | X | X |
| HAQ/VAS | X | X | X | X |
| PASI/BSA | X | X | X | X |
| synovial biopsy |  | X | X |  |
| skin biopsy |  | X | X |  |
| PBMCs |  | X | X |  |
| adverse events |  | X | X | X |
| concomitant medication | X | X | X | X |

**CONTENT**

1. Introduction Psoriatic arthritis and treatment

Synovial biopsy and immunohistochemistry

Skin biopsy and immunohistochemistry

2. Study objectives Primary objective

Secondary objectives

3. Investigational plan Study medication and dosage

Design

Statistical base for sample size

Patient identification

Duration of study

Selection of study patients

*Inclusion criteria*

*Exclusion criteria*

*Withdrawal and replacement of patients*

Variables and schedule of assessments

*Background information*

*Efficacy variables*

*Primary variables*

*Secondary efficacy variables*

### Safety variables

*Laboratory variables*

*Clinical variables*

Concomitant illnesses

Concomitant treatment

Adverse events

*Adverse event severity and reporting*

4. Methods of investigation Efficacy criteria

Statistical methods

# 5. Ethical and legal aspects Good clinical practice

Informed consent of patient

Approval of study protocol

Confidentiality

Liability and insurance

Use of study findings

6. Amendments

References

## APPENDICES

APPENDIX 1 Health Assessment Questionnaire (Dutch version)

APPENDIX 2 Visual Analogue Scale (VAS) pain (Dutch version)

VAS disease activity (Dutch version)

Patient global assessment (Dutch version)

Physician global assessment (Dutch version)

APPENDIX 3 Tender joint count, swollen joint count

APPENDIX 4 Standard Operating Procedure biopsy handling

APPENDIX 5 Psoriasis Area and Severity Index (PASI)

**1. INTRODUCTION**

**Psoriatic arthritis and treatment**

Psoriatic arthritis (PsA) occurs in up to 30% patients with psoriasis [1] and is a common chronic rheumatic disease that may result in considerable joint damage if left untreated [2]. Early aggressive treatment is indicated in patients with significant joint inflammation. Conventional therapeutic regimens with disease modifying anti-rheumatic drugs (DMARDs) in psoriatic arthritis are often based on clinical experience or extrapolated data from studies in rheumatoid arthritis (RA), rather than clinical trial evidence in PsA. Currently, sulphasalazine, methotrexate, and leflunomide are considered to be the DMARDs of choice for arthritis in PsA, but the clinical benefit of these DMARDs is marginal to modest [3]. In PsA patients a high concentration of TNF-α, a proinflammatory cytokine, is present in synovial fluid and synovial tissue, as well as in psoriatic skin [4-6]. TNF-α has proven to be an effective new target for therapy in both psoriasis and PsA [7] .

Our previous work has shown that anti-TNF therapy induces marked beneficial changes at the site of inflammation early after initiation of treatment, both in RA [8,9] and in psoriatic arthritis [10,11].

##### Synovial and skin biopsy in clinical trials

There has been great interest in synovial tissue for the evaluation of disease activity in several forms of arthritis, especially in the research aimed at the development of new treatments. Improvement of the techniques has made the synovial biopsy a safe and uncomplicated procedure, which can be done under local anesthesia in the outpatient clinic [12]. Using a (mini-)arthroscopy sites of active and non-active inflammation can be discriminated and adequate tissue can be obtained in nearly all cases.

Synovial biopsies can be used to evaluate the mode of action of treatment in relation to treatment response using a variety of techniques [13]. Analysis of synovial biopsies for the expression of several markers (e.g. to quantify the cellular infiltrate, pro-inflammatory cytokines, adhesion molecules) The laboratory of the Division of Clinical Immunology and Rheumatology of the Academic Medical Center, has extensive experience with these techniques and has developed an elaborate quality control system for clinical trials, immunohistologic analysis, and computer-assisted digital image analysis [14-16].

Similarly, skin biopsies can be obtained easily under local anesthesia from a psoriatic plaque and can be used to evaluate changes in histologic and immunohistochemical features. Of specific interest will be the assessment of the phenotype of T cells and other inflammatory cells. There is extensive experience with these techniques at the Department of Dermatology of the Academic Medical Center and both departments have a longstanding collaboration in the evaluation of the effects of targeted therapies in patients with psoriasis and psoriatic arthritis [10,11,17,18].

**Examination of the tissue response to treatment to predict clinical effects.**

It can be anticipated that rapid developments in immunology, molecular biology, and biotechnology will lead to a dramatic increase in the number of new, targeted therapies. Therefore, there is a clear need for biomarkers, which could be used for selection purposes during the development process. Examination of serial synovial samples has been proposed as a method that can be used to predict a potential effect of novel antirheumatic interventions [19]. Successful treatment with corticosteroids as well as DMARDs, such as gold, methotrexate and leflunomide, is associated with decreased mononuclear cell infiltration. Similarly, successful treatment of RA subjects with for infliximab and etanercept results in reduced synovial inflammation [13-15]. The effects of adalimumab have not been studied previously.

Of importance is the fact that analysis of serial synovial samples from RA subjects who received either placebo or unsuccessful treatment with recombinant human interleukin-10, IFN-beta, and anti-CD4 antibodies did not reveal significant changes in key synovial biomarkers [15,20-22]. Similarly, there was no clear-cut change in serial biopsies after treatment with IL-1 receptor antagonist at 30 mg/day [23], which appears to have very limited effects on arthritis activity. Taken together, these studies indicate that analysis of serial biopsies can be used as a screening method to test new compounds requiring relatively small numbers of subjects. The absence of changes after treatment would suggest that the therapy is probably not effective. This approach has recently been successful in the evaluation of effects of novel therapeutic strategies for RA, but has as yet not been systematically validated in psoriatic arthritis.

# 2. STUDY OBJECTIVES

**Primary objective**

The primary objective is to study changes in synovial and skin inflammation and cytokine expression in serial biopsy samples following administration of adalimumab therapy to subjects with active psoriatic arthritis.

# Secondary objectives

The secondary objectives of this study are to (i) assess clinical response (ii) compare immunohistochemical analysis and Polymerase Chain Reaction (PCR) analysis of cytokines in tissue samples (iii) to assess changes in gene expression in synovial and skin tissue by microarray analysis (iv) to assess changes in serum biomarkers

# 3. INVESTIGATIONAL PLAN

## **Study medication and dosage**

## Trade name: Humira® or matching placebo

Dosage: 40 mg every other week by subcutaneous injection

The study medication will be supplied by the sponsor of this study for the first 4 weeks (first 2 injections).

The investigators will administer/dispense the study medication only to patients included in this study following the procedures set out in the study protocol.

## **Design**

Following a screening period of 2 weeks, patients with active PsA will be enrolled into a (1 : 1) 4-week randomized double-blind, placebo-controlled treatment period. After the second biopsy (at 4 weeks), all patients will receive adalimumab (Humira®). Patients are allowed to use concomitant NSAIDs, corticosteroids (prednisone equivalent ≤ 10 mg/day) and methotrexate, provided the dose has been stable for at least 4 weeks prior to baseline.

Biopsies of one target skin lesion will be obtained before administration (preferable from a sun-protected area) of study drug at baseline, and after 4 weeks of treatment (a total of 4 skin biopsies). Synovial biopsies of a clinically inflamed joint (knee, wrist or ankle) will be obtained by arthroscopy before administration of study drug and after 4 weeks of treatment (a total of 2 synovial biopsies). The baseline visit and first arthroscopy *must* be within 3 days before the first administration of study medication. After 4 weeks the arthroscopy will be repeated in the same joint.

A clinical evaluation of both joint and skin symptoms will be repeated after 4 and 12 weeks of treatment. Patients will be seen for efficacy and safety assessments in accordance with standard guidelines for clinical practice during the entire study.

In total there will be 4 study visits: screening, baseline, week 4 and week 12.

There will be a ± 3-day deviation for all return visits. All visits will be fixed with reference to the baseline visit.

### **Statistical basis for sample size**

Only limited knowledge exists on the primary variables used in this study, i.e. the immunohistochemical changes in synovial tissue and skin. Therefore, a formal estimate of the sample size is not possible. Twenty-four male and female subjects with psoriatic arthritis will be recruited to the study, based on our previous experience in comparable studies in RA [14,15] and psoriatic arthritis [10,11,17,18]. The number of subjects is based upon the desire to gain adequate information, while exposing as few subjects as possible to study procedures. Information gained from this study will be used to power future studies.

# Patient identification

All patients must be identifiable throughout the study, even during the run‑in phase (screening to baseline). Therefore, each patient entering the study at screening visit will be assigned a *screening number* (A-01, A-02, A-03, A-04, etc.). Patients will be numbered consecutively; the first patient being screened will receive number A-01, the second patient A-02 and so on. The results of screening procedures will be recorded under this screening-number. On completion of the run‑in phase, all eligible patients will receive a treatment number, a number that might not be identical with the screening number.

Example:

screening number treatment number

subject 1 A-01 B-01

subject 2 A-02 B-02

subject 3 A-03 (not eligible)

subject 4 A-04 B-03

subject 5 A-05 B-04

Patients who are withdrawn from the study for whatever reason will be replaced. Patient screening will be stopped when a total of 24 patients has entered the study.

### **Duration of study**

Duration of the study for the individual subject will be 12 weeks, preceded by a screening period of 2 weeks. The study will be finished after 12 weeks of treatment. Patients who respond well to therapy will be allowed to continue treatment with Humira® in a normal outpatient setting with their own rheumatologist.

## **Selection of study patients**

A patient may be enrolled in this study provided that he/she has met all of the inclusion criteria and none of the exclusion criteria. The patients with a diagnosis of active psoriatic arthritis will be recruited at the Division of Clinical Immunology and Rheumatology and the Department of Dermatology of the Academic Medical Center in Amsterdam.

### Inclusion criteria

1. Prior to any study procedure, voluntary written informed consent must be obtained, after the nature and purpose of this study are explained
2. Patients should be between 18 and 80 years of age
3. Patients have presence of active cutaneous lesions of psoriasis or documented history of psoriasis diagnosed by a dermatologist
4. Patients must have a diagnosis of psoriatic arthritis for at least 3 months. The disease must be moderate to severely active as defined by ≥ 2 swollen and ≥ 2 tender or painful joints
5. If female, patient should either be of not-childbearing potential (i.e. postmenopausal or surgically sterile) or practice a reliable method of birth control until 150 days post-study (e.g. use of condom, IUD, oral contraceptives) or have a vasectomized partner
6. Patients should have inadequate response to NSAID therapy
7. The use of concomitant NSAIDs and corticosteroids is allowed. The dose of corticosteroids should not exceed a prednisone equivalent ≤ 10 mg/day and must be stable for at least 4 weeks prior to baseline
8. The use of concomitant methotrexate (MTX) is allowed. If using MTX, patients must have received a minimum of 3 months of therapy and be on a stable dose for at least 4 weeks prior to baseline
9. Patients are considered to be in generally good health based upon the result of a medical history, physical examination, laboratory profile, chest  X-ray and ECG

### Exclusion criteria

Patients will be considered ineligible for study participation is any of the following criteria are met:

1. Patient has previously received anti-TNF therapy, or another investigational drug for psoriasis or psoriatic arthritis **in the past 2 months**
2. Patient has received any DMARD or systemic psoriasis therapy (other than MTX) within 4 weeks prior to baseline
3. Patient has received an intra-articular injection with corticosteroids within 4 weeks prior to baseline
4. Patient has an active skin or connective tissue disease (other than psoriasis and psoriatic arthritis) that would interfere with the assessment of either psoriasis or arthritis
5. Patient has received topical psoriasis therapy (e.g. corticosteroids, vitamin D analogues, keratolytics, coal tar or topical retinoids) within 2 weeks prior to baseline

NB: low potency topical steroids to be used for scalp, palms, groin and/or soles of feet only are allowed

1. Patients has a history of active tuberculosis. A PPD test and chest X-ray done at screening should be negative (in case of latent tuberculosis, a patient may enter the study if prophylaxis with isoniazide is begun prior to administration of adalimumab)
2. Patients has a recent history of (or persistent) infection requiring hospitalization or antibiotic treatment within 4 weeks of baseline
3. Patient is HIV positive. A HIV test done at screening has to be negative
4. Patient has evidence of a hepatitis B or hepatitis C virus infection. A serologic test for HBV and HCV done at screening has to be negative
5. Patient has a significant history of cardiac, renal, neurological, metabolic or any other disease that may affect his/her participation in this study
6. Patient has a history of malignancy (other than basal cell carcinoma of the skin) in the past 10 years
7. If female, patient should not be pregnant or breast-feeding. A serum pregnancy-test will be performed at screening has to be negative
8. Patient is, in the opinion of the investigator, unable to comply with the requirements of the study protocol or is unsuitable for the study for any reason

Any patient who does fulfill the inclusion criteria at screening, but not at baseline may be screened again, and if inclusion criteria are met, enter the study.

### Withdrawal and replacement of patients

Patients may be withdrawn for the following reasons:

- at their own request
- at the discretion of the investigator

Patients must be withdrawn under the following circumstances:

- in case of any intercurrent illness or adverse event, that interferes with the safety of continuous administration of study-drug to the patient

In all cases, the reasons why patients are withdrawn must be recorded in detail in the patient's medical records and the CRF. Patients that leave the study or are withdrawn from the study before week 4 will be replaced.

## **Variables and schedule of assessments**

The following will be determined in accordance with the "Schedule of assessments".

### Background information

At the screening visit of the study, all patients will undergo a full physi­cal examination, and the relevant medical history will be taken. Further to this demographic data will be collected/measured and documented.

The following data will be documented:

. date of obtaining informed consent

. initials, date of birth, sex, race, height, weight

. alcohol consumption/smoking habits

. concomitant illnesses

. concomitant medication

. psoriatic arthritis disease status and time since diagnosis

. prior DMARD history

. relevant findings in physical examination

### Efficacy variables

#### *1. Primary variables*

Primary variables will include the analysis of the cell infiltrate and the expression of cytokines. At baseline an arthroscopy with synovial biopsies of an inflamed joint (knee, wrist or ankle) will be performed. The arthroscopy and biopsies will be repeated in the same joint 4 weeks after first administration of the study drug and before the third injection. Immunohistochemical staining will be performed and analyzed by digital image analysis, as we have previously described and validated for synovial tissue [16].

Initially scores will be obtained for each of the following items:

1. for analysis of the cellular infiltrate:

- CD3 (T-cell marker)
- CD4
- CD8
- CD22 (B-cell marker)
- CD68 (macrophages)
- CD163
- MRP8
- MRP14
- CD38 (plasma cells)
- CD55 (fibroblast like synoviocytes)
- granzyme B (cytotoxic cells)
- CD15

1. for analysis of cytokine expression:

- IL-1β
- IL-6
- TNF-α

1. for analysis of MMP expression:

- MMP-1
- MMP-3
- MMP-13

1. for analysis of vascularity

- Factor VIII

e) Tissue samples will be stored for future microarray analysis

In addition, PBMCs will be collected before treatment and 4 weeks after the initiation, and serum will be collected before treatment and 4 and 12 weeks after the initiation to determine changes in biological markers.

*2. Secondary efficacy variables*

Secondary variables will consist of:

- Skin biopsies (in total 4x 4 mm punch biopsies) will be performed at baseline and 4 weeks after first administration of study drug from the same lesion. 2 adjacent biopsies will be obtained at each time point from the edge of an active lesion. One of the biopsies will be embedded in tissue-tek before the freezing process for cryosectioning. The other biopsy will be placed in a cryotube and directly frozen in liquid nitrogen for possible PCR or microarray analysis in the future.

The following analysis will be performed:

a) epidermal thickness and morphology

1. assessment of T cells and their activity:

-CD3 (T cell marker)

-CD25 (T cell marker)

1. other inflammatory cells:
   - CD15 (neutrophil marker)
   - CD68 (macrophage marker)
   - CD83 or DC-LAMP (dendritic cell markers)
2. proinflammatory cytokines

- TNF-α
- IFN-γ
- The clinical severity of the skin lesions during treatment will be evaluated at each visit using the following parameters:

- Psoriasis Area and Severity Index (PASI) (see appendix)

- total affected Body Surface Area (BSA)

- The clinical severity of the affected joints during treatment will be evaluated at each visit using the following parameters:

-Tender joint count (see appendix)

- Swollen joint count (see appendix)

-Visual Analogue Scale (VAS) pain (see appendix)

-VAS disease activity (see appendix)

-CRP and ESR

-Health Assessment Questionnaire (see appendix)

-Physician’s Global Assessment of Disease (see appendix)

-Patient’s Global Assessment of Disease (see appendix)

### Safety variables

#### *1. Laboratory variables*

HEMATOLOGY: hemoglobin, white blood cell count, platelet count, ESR

chemistry: creatinine, alkaline phosphatase, ASAT, ALAT, LDH, CRP

URINALYSIS: urine dipstick

The following laboratory variables will be assessed at screening only:

- Chest X ray

- PPD skin test

- Virology: tests for HIV, HCV and HBV

- Pregnancy test: serum β-hcg in women of child-bearing potential

- Rheumatoid factor, anti-CCP

In this study per patient a total volume of approximately 70-ml of blood will be drawn for study purposes over a period of 14 weeks.

#### *2. Clinical variables*

As a clinical safety monitoring the following examinations will be performed:

- A complete medical history will be taken at enrollment (screening visit).
- Physical examination will be performed at screening visit and at the end of the treatment period (week 12).

Any evaluations that are abnormal and considered to be related to the study medication will be followed until they return to baseline or are judged not to be clinically significant.

### **Concomitant illnesses**

Other illnesses present at entry to the study are regarded as con­comitant illnesses and will be documented on the case record form pro­vided. Any concomitant illness such as diabetes, hypertension, heart failure etc. should be controlled before entry into the study by appropriate medication which should, if possible, not be changed during the entire study period.

Concomitant illnesses, relevant medication and any changes in concomitant disorders and/or medication have to be documented appropriately in the case record form (CRF).

Illnesses occurring during the study period are to be regarded as adverse events and will each be documented on a separate "Adverse event" page in the case record form. Application of study medication may be continued during intercurrent illnesses, if clinically justifiable.

### **Concomitant treatment**

All treatments being taken by the patients on entry to the study and all treatments given in addition to the study treatment during the study are regarded as concomitant treatments and must be docu­­mented on the case record form, as mentioned before.

The following concomitant treatments (for psoriatic arthritis) are permitted during this study, provided that the patients have received a stable dose for at least 28 days before entering the study:

- nonsteroidal anti-inflammatory drugs (NSAIDs) including acetylsalicylic acid
- oral corticosteroids, if not exceeding a prednisone equivalent of 10 mg daily
- methotrexate

The following concomitant treatments are not permitted during this study (see exclusion criteria):

- intra-articular corticosteroid injections
- oral steroids, if exceeding a prednisone equivalent of 10 mg daily
- DMARDs other than methotrexate
- topical corticosteroids, vit.D analogues, topical retinoids, keratolytics or coal tar (other than on the scalp, palms, groin, and/or soles of feet).

It is important that steroid treatment be kept to a minimum. Changes of NSAIDs and increases in steroid therapy (drug or dosing) should be avoided. If there is a clear reason to do so, the reason and the changes have to be documented in detail in the case record form.

If therapy for pain relief becomes necessary throughout the study, acetaminophen should be used and has to be documented in detail in the case record form. No pain medication should be used within 6 hours prior to joint examination or arthroscopy. Opiates should be avoided.

Patients requiring surgical procedures involving local or regional anesthesia may continue with study medication. Surgical procedures involving general anesthesia should preferably be postponed until the end of the study, if possible. Should patients require (major) surgery involving general anesthesia during the 12-week study period, study medication will be stopped an patients are withdrawn from the study.

## **Adverse events**

An adverse event is defined as any untoward medical occurrence in a clinical investigation subject administered a pharmaceutical product. The term adverse event does not imply a causal re­lation­ship with the study treat­ment.

The period of observation for adverse events extends from the time the patient gives informed consent until he or she undergoes the final exami­nation as part of the study. Adverse events occurring after this period should also be mentioned in the CRF, if the adverse event comes to the knowledge of the investigator.

All adverse events ‑ whether considered as­soci­ated with the use of the study medication or not ‑ must be followed to a satisfactory conclusion, i.e. until they return to baseline, become stabilized or until there is a satis­factory explanation for the changes ob­served. In case of death a full pathologist's re­port should be supplied, if possible. All findings must be reported in the case record form and in the patient's medi­cal re­cords.

### Adverse event severity and reporting

An adverse event is considered a serious adverse event (SAE) if it meets the following criteria:

- any event that is fatal or life‑threatening
- any event that is permanently or significantly disabling
- any event that requires or prolongs hospitalization
- any event that involves cancer or congenital anomaly
- any event that occurs with overdose (any dosage higher than that recommended in the Investigator's Brochure or package insert)
- any event that suggests a significant hazard

All serious adverse events which occur during the period of observation MUST be re­ported within 24 hours or at the latest on the following working day by tele­phone and/or telefax to the sponsor, whether consid­ered to be associated with the study medication or not. The event will be docu­mented in the CRF, as complete as possible. If considered necessary, the inves­tigator will also in­form the local medical ethics committee and authorities.

Any adverse events considered not to be a serious adverse event, should be documented in the CRF.

Regardless of the classification of an adverse event as serious (see above), the severity of all adverse events must be assessed as mild, moderate or se­vere.

*severe* the adverse event causes considerable interference with the subject’s usual activities and may be incapacitating or life-threatening

*moderate* the adverse event causes the subject discomfort and interrupts the subject’s usual activities

*mild* the adverse event is transient and easily tolerated by the subject

Furthermore, the investigator will assess the relationship of the adverse event to the use of the study drug into:

*not related* adverse event is due to an underlying or intercurrent illness or effect of another drug and is not related to the study drug

*probably not related* adverse event has little temporal relationship to study drug and a more likely etiology of the adverse event exists

*possibly related* adverse event has a strong temporal relationship to the study drug and an alternative etiology is less likely

*probably related* adverse event has a strong temporal relationship to the study drug or recurs on re-challenge and another etiology is unlikely

If in the opinion of the investigator the adverse event is considered (probably) not related, an alternative etiology must be provided.

# 4. METHODS OF INVESTIGATION

The clinical examinations including vital signs as well as safety assessments will be performed as usual in routine clinical practice.

The procedure for arthroscopy and biopsy handling to obtain tissue samples for the immunohistochemical assessments will be outlined in Appendices, which will be affixed to this protocol. The studies of synovial tissue will be performed at the Laboratory for Experimental Immunology/Division of Clinical Immunology and Rheumatology, Academic Medical Center, Amsterdam, The Netherlands. The studies of lesional skin will be performed at the Experimental Laboratory for Dermatology, Academic Medical Center, Amsterdam, The Netherlands.

Routine laboratory (hematology, chemistry and urinalysis as mentioned before) will be performed at all 4 study visits. Furthermore, blood will be drawn at the 2 arthroscopy visits at baseline and week 4 (3 heparin tubes, 1 serum tube, 1 EDTA tube at each time point). Mononuclear cells (PBMCs) will be isolated and stored in liquid nitrogen. Serum and plasma will be immediately frozen at -70°C. The material will be used for detection of biologic markers (such as cytokines), leukocyte differential, and gene activation (micro-array) in relation to the disease. All samples must be clearly labeled (using labeling that can withstand freezing in liquid nitrogen/dry ice) including patient number (and patient’s date of birth) and date and time of sampling.

## **Efficacy criteria**

#### *Analysis of the cell infiltrate and expression of mediators of inflammation of synovial tissue (and lesional skin)*

The effects of adalimumab compared to placebo on immunohistochemical indices for synovial inflammation and psoriatic skin lesions will be assessed on biopsies obtained by arthroscopy and by skin biopsies (4mm punch biopsy) from a target lesion.

## **Statistical methods**

The evaluation will comprise a description of the study population, a comparison of baseline values and the changes in the synovial features and clinical parameters after treatment with adalimumab for 4 weeks. The clinical evaluation after 12 weeks of treatment will be used to compare clinical response at 12 weeks to the synovial tissue response at 4 weeks. In addition, we will describe the clinical effects at each time point in all patients.

The findings will be tested for normal distribution. If this requirement is not fulfilled, appropriate non-parametric methods will be used. All statistical tests will be two-tailed and performed at the 5% significance level.

In addition, each of the end points will be statistically analyzed using an analysis of covariance (ANCOVA) model. The model included terms for treatment as a fixed effect and the baseline measurement as a covariate. The aim is to assess the treatment difference.

# 5. ETHICAL AND LEGAL ASPECTS

## **Good clinical practice**

The procedures set out in this study protocol are designed to ensure that the sponsor and the investigator abide by the principles of the good clinical practice guidelines of the European Community and the Declaration of Helsinki in the conduct, evaluation and documentation of this study. The study will also be carried out in keeping with local legal requirements.

## **Informed consent of patient**

Before undergoing any study procedure, the patient must have given his/her written informed consent to participate after the nature, scope and possible con­sequences of the clinical study have been explained in an understandable way. The patient will also be given an information sheet about the study and a copy of the signed informed consent form. The terms of the consent and when it was obtained must also be documented in the case record form.

## **Approval of study protocol**

Before the start of the study, the study protocol and/or other appropriate documents will be submitted to the ethics committee and/or the authori­ties, in accordance with local legal require­­ments.

## **Confidentiality**

All patient names will be kept secret. The number al­lotted to them during the study will identify patients throughout documentation and evaluation. The patients will be told that all study findings will be stored and handled in strictest confidence.

The signed informed consent forms remain with the investigator. By signing the declaration in Section 10 of this study protocol, the investi­gator agrees to obtain a correctly completed informed consent form for each patient included in the study. He/she also agrees to allow these to be inspected on request. The investiga­tor will maintain a personal list of patient numbers and patient names to enable records to be found at a later date. Both the consent forms and the personal list of patient numbers will be kept for 15 years.

## **Liability and insurance**

The civil liability of the investigator, the persons instructed by him/her and the hospital, practice or institute in which they are employed and the li­ability of AMC Medical Research B.V. (AMR) in respect of financial loss due to personal injury and other damage which may arise as a result of the carrying out of this study are governed by the applicable law.

The AMR has taken out reasonable third-party liability insurance cover. As a precautionary measure, the investigator, the persons instructed by him/her and the hospital, practice or institute are included in such cover in respect of work done by them in carrying out this trial to the extent that the claims are not covered by their own professional indemnity insur­ance.

Such insurance covers claims provided that:

- the instructions and procedures laid down in the study protocol are followed precisely and the investigator acts with the necessary care
- the patients do not undergo any other medical treatment without the previous consent of the investigator and that the investigator has in­formed patients to that effect
- the patient informs the investigator immediately of any unforeseeable adverse events
- the physical injury or other damage does not arise as a result of any gross negligence, willful act or omission

## **Use of study findings**

By signing the study protocol, the investigator agrees with the use of results of the study for the purposes of national and international registra­tion, publication and information for medical and pharmaceutical professionals. If necessary, the authorities will be notified of the investigator­'s name, address, qualifications and extent of involvement.

The findings of this study may be published in a scientific jour­nal and pre­sented at scientific meetings.

# 6. PROTOCOL AMENDMENTS

Amendments should be made only in exceptional cases once the study has star­ted. Changes must be agreed to in writing, and signed, by all parties concerned. The changes then become part of the study protocol.

The medical ethics committee must be informed of all amendments and if necessary approval must be sought for ethical aspects. Approval must also be obtained from the authorities if necessary.

**APPENDIX 1**

| **VRAGENLIJST TER BEOORDELING VAN DE GEZONDHEID (HAQ)** | | | | |  | | | | | | | |
| --- | --- | --- | --- | --- | --- | --- | --- | --- | --- | --- | --- | --- |
| **Initialen**______________________________________**Datum**_____________________________ | | | | |  | | | | | | | |
| Met deze vragen willen we te weten komen hoe uw ziekte uw dagelijks functioneren beïnvloedt. Eventuele opmerkingen kunt u aan de achterzijde van deze bladzijde opschrijven.  **Kruis a.u.b. het antwoord aan dat het beste weergeeft waartoe u IN DE AFGELOPEN WEEK doorgaans in staat bent geweest:** | | | | |  | |  | |  | |  | |
| **AANKLEDEN & PERSOONLIJKE VERZORGING**  Was u in staat om: | Zonder enige  moeite | Met  ENIGE  moeite | Met  VEEL  moeite | Niet in staat  om het  te doen |  | | | | | | | |
| - Uzelf aan te kleden, met inbegrip van schoenveters strikken en knopen dichtdoen? |  |  |  |  |  | | | | | | | |
| - Uw haar te wassen? |  |  |  |  |  | | | | | | | |
|  | | | | |  |  | |  | |  | |  |
| **OVEREIND KOMEN**  Was u in staat om: |  |  |  |  |  | | | | | | | |
| - Op te staan uit een rechte stoel zonder leuningen? |  |  |  |  |  | | | | | | | |
| - In en uit bed te komen ? |  |  |  |  |  | | | | | | | |
|  | | | | |  |  | |  | |  | |  |
| **ETEN**  Was u in staat om: |  |  |  |  |  | | | | | | | |
| - Uw vlees te snijden? |  |  |  |  |  | | | | | | | |
| - Een vol kopje of glas naar uw mond te brengen? |  |  |  |  |  | | | | | | | |
| - Een onaangebroken pak melk te openen? |  |  |  |  |  | | | | | | | |
|  | | | | |  |  | |  | |  | |  |
| **LOPEN**  Was u in staat om: |  |  |  |  |  | | | | | | | |
| - Buitenshuis op vlakke grond te lopen? |  |  |  |  |  | | | | | | | |
| - Vijf treden op te lopen? |  |  |  |  |  | | | | | | | |
|  |  |  |  |  |  | | | | | | | |
| **Kruis a.u.b. elk hulpmiddel of gebruiksvoorwerp aan dat u gewoonlijk bij bovengenoemde bezigheden gebruikt:**   Wandelstok  Hulpmiddelen bij het aankleden (haakje om knopen dicht te doen of om ritsen te sluiten, schoenlepel met verlengd handvat, etc.)   Looprek of rollator  Speciaal aangepaste gebruiksvoorwerpen   Krukken  Speciaal aangepaste stoel   Rolstoel  Anders (te weten:___________________________) | | | | |  | | | | | | | |
| **Kruis a.u.b. alle bezigheden aan waarbij u gewoonlijk hulp van iemand anders nodig heeft:**   Aankleden en persoonlijke verzorging  Eten   Overeind komen  Lopen | | | | |  | | | | | | | |

| **Kruis a.u.b. het antwoord aan dat het beste weergeeft waartoe u IN DE AFGELOPEN WEEK doorgaans in staat bent geweest:** | | | | |  |  |  |  |
| --- | --- | --- | --- | --- | --- | --- | --- | --- |
| **HYGIËNE**  Was u in staat om: | Zonder enige  moeite | Met  ENIGE  moeite | Met  VEEL  moeite | Niet in staat  om het  te doen |  | | | |
| - Uw hele lichaam te wassen en af te drogen? |  |  |  |  |  | | | |
| - Een bad te nemen? |  |  |  |  |  | | | |
| - Op het toilet te gaan zitten en er weer vanaf te komen? |  |  |  |  |  | | | |
| **BEREIK**  Was u in staat om: |  |  |  |  |  | | | |
| - Een voorwerp van ± 2 kilo (bijv. een pak waspoeder) van een plek vlak boven uw hoofd te pakken en neer te zetten? |  |  |  |  |  | | | |
| - Te bukken om kleding van de vloer op te rapen? |  |  |  |  |  | | | |
| **GRIP**  Was u in staat om: |  |  |  |  |  | | | |
| - Autodeuren te openen? |  |  |  |  |  | | | |
| - Potten te openen die al een keer open gemaakt zijn? |  |  |  |  |  | | | |
| - Kranen open en dicht te draaien? |  |  |  |  |  | | | |
| **ACTIVITEITEN**  Was u in staat om: |  |  |  |  |  | | | |
| - Boodschappen te doen en te winkelen? |  |  |  |  |  | | | |
| - In en uit een auto te stappen? |  |  |  |  |  | | | |
| - Karweitjes te doen zoals stofzuigen of tuinieren? |  |  |  |  |  | | | |
| **Kruis a.u.b. elk hulpmiddel of gebruiksvoorwerp aan dat u gewoonlijk bij bovengenoemde bezigheden gebruikt:**    Verhoogde WC-bril  Handgreep in bad   Zitje in het bad  Gebruiksvoorwerpen met een verlengd handvat om dingen te kunnen pakken    Pot-opener (voor potten die  Gebruiksvoorwerpen met een verlengd   al eens geopend zijn) handvat voor in de badkamer (bijv. een borstel).   Anders (te weten:__________________________) | | | | |  | | | |
| **Kruis a.u.b. alle bezigheden aan waarbij u gewoonlijk hulp van iemand anders nodig heeft:**   Hygiëne  Grip en het openen van dingen   Bereik  Boodschappen en huishoudelijke karweitjes | | | | |  | | | |
|  | | | | |  | | | |

[*Zandbelt MM, Welsing PM, van Gestel AM, van Riel PL.*](http://www.ncbi.nlm.nih.gov:80/entrez/query.fcgi?cmd=Retrieve&db=PubMed&list_uids=11502610&dopt=Abstract) *Health Assessment Questionnaire modifications: is standardisation needed? Ann Rheum Dis. 2001 Sep;60(9):841-5.*

**APPENDIX 2**

**VAS algehele ziekteactiviteit**

Hoe voelt u zich als u alle aspecten van de reuma (zoals ochtendstijfheid, pijn, zwelling, en beperkingen in het algemeen) laat meewegen?

*Wilt u een verticaal streepje zetten op onderstaande lijn?*

### Zeer goed _____________________________________________Zeer slecht

###

0 mm 100 mm

**VAS pijn**

Hoeveel pijn heeft u IN DE AFGELOPEN WEEK vanwege uw ziekte gehad?

*Wilt u een verticaal streepje zetten op onderstaande lijn?*

### Helemaal ___________________________________________ ___ Extreem

### geen pijn veel pijn

0 mm 100 mm

**Evaluatie algehele ziekteactiviteit (patiënt)**

Beoordeling **patiënt** van zijn/haar algehele ziekteactiviteit:

Hoe voelt u zich als u alle aspecten van de reuma (zoals ochtendstijfheid, pijn, zwelling, en beperkingen in het algemeen) laat meewegen?

1 = erg goed

2 = goed

3 = redelijk

4 = slecht

5 = erg slecht

**Evaluatie algehele ziekteactiviteit (onderzoeker)**

Beoordeling PsA door **onderzoeker**:

1 = erg goed

2 = goed

3 = redelijk

4 = slecht

5 = erg slecht

*Felson DT, Anderson JJ, Boers M, Bombardier C, Furst D, Goldsmith C et al. American College of Rheumatology preliminary definition of improvement in rheumatoid arthritis. Arthritis Rheum 1995; 38:727-735.*

**APPENDIX 3 68-JOINT SCORE**

|  | **Right** | | **Left** | |
| --- | --- | --- | --- | --- |
|  | Pain | Swelling | Pain | Swelling |
| Shoulder |  |  |  |  |
| Elbow |  |  |  |  |
| Wrist |  |  |  |  |
| MCP-1 |  |  |  |  |
| MCP-2 |  |  |  |  |
| MCP-3 |  |  |  |  |
| MCP-4 |  |  |  |  |
| MCP-5 |  |  |  |  |
| IP-1 |  |  |  |  |
| PIP-2 |  |  |  |  |
| PIP-3 |  |  |  |  |
| PIP-4 |  |  |  |  |
| PIP-5 |  |  |  |  |
| DIP-2 |  |  |  |  |
| DIP-3 |  |  |  |  |
| DIP-4 |  |  |  |  |
| DIP-5 |  |  |  |  |
| Hip |  |  |  |  |
| Knee |  |  |  |  |
| Ankle |  |  |  |  |
| MTP-1 |  |  |  |  |
| MTP-2 |  |  |  |  |
| MTP-3 |  |  |  |  |
| MTP-4 |  |  |  |  |
| MTP-5 |  |  |  |  |
| IP-1 |  |  |  |  |
| PIP-2 |  |  |  |  |
| PIP-3 |  |  |  |  |
| PIP-4 |  |  |  |  |
| PIP-5 |  |  |  |  |
| DIP-2 |  |  |  |  |
| DIP-3 |  |  |  |  |
| DIP-4 |  |  |  |  |
| DIP-5 |  |  |  |  |
|  |  |  |  |  |
| TOTAL |  |  |  |  |

0 = no tenderness

1 = pain on pressure

2 = pain and winced

3 = winced and withdrew

9 = not done, not assessable

Initialen v.d onderzoeker I_I_I_I Datum I_I_I-I_I_I_I-I_I_I

*Dag Maand Jaar*

**APPENDIX 4**

Standard Operating Procedure (SOP) biopsy handling Date: 11-09-2002

### Author : P.Blankert/ T.Smeets

### Version : 2 ____________________________________________________

- The arthroscopist collects at least 24 samples for the different analysis procedures.


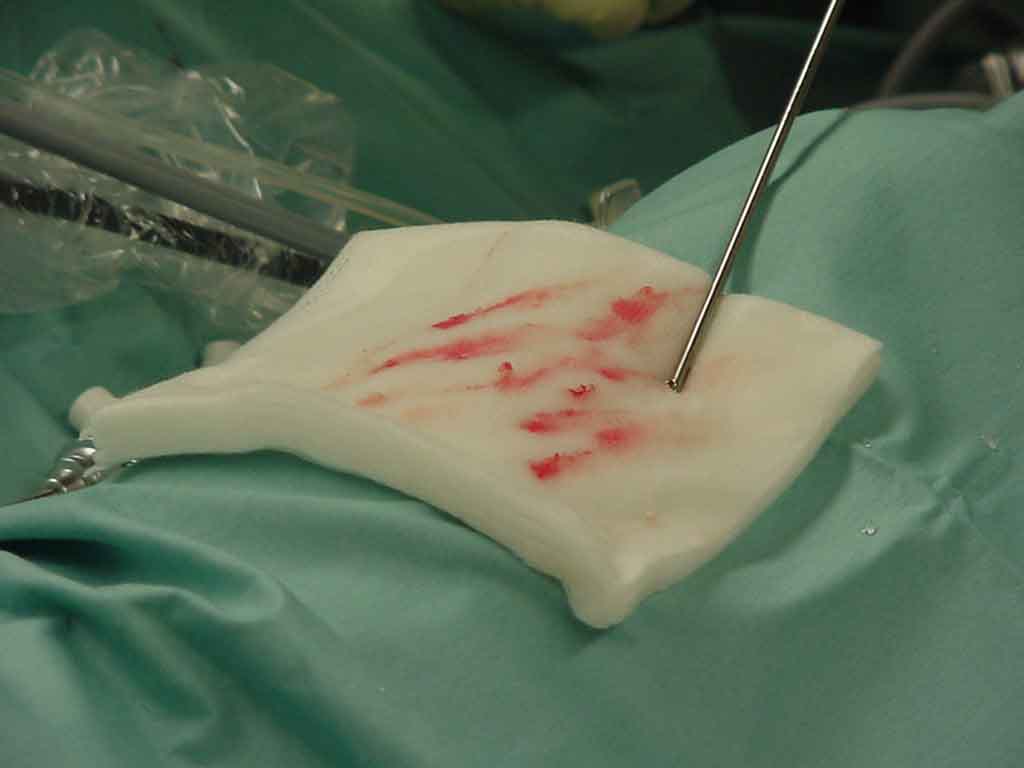


For each study, a detailed collection scheme is available in the study specific file.

- Each set of 6 samples is collected on a wet gauze (saline 0.9%) and directly processed by the technician.

- Cryotubes are labeled with a waterproof lumicolor the following way:

Patient ID (Bx…..),procedure (IHC,ISH,PCR, MA or EMSA), date of the arthroscopy.

- Tubes with formaline for pathology are available at the arthroscopy room (top drawer of the table) and labeled as follows:

Patient name

AMC number

Date

- Tubes for EM are stored in the +4°C room (G1-125) bottom shelf of “reuma corner” (or available at pathology) and labeld as follows:

Study

Bx number

AMC number

Arthroscopy number

- Tubes for “lyme”disease are stored at -20°C (reuma) outside G1-111 in the corridor (or available at bacteriology) and labeled as follows:

Patient name

AMC number

Date

- Tubes for TBC / bacterial culture are stored at +4°C room (G1-125) bottom shelf of “reuma corner” (or available at bacteriology) and labeled as follows:

Patient name

AMC number

Date

After the arthroscopy :

- **PA**-tubes are given to the nurse
- **EM**-tubes are stored in the rheumatology case in the corridor of G1-111.
- **Lyme disease**-tubes are taken to bacteriology with a form by us or the nurse
- **TBC / Culture**-tubes are taken to bacteriology with a form by us or the nurse
- **Step 1. for IHC and ISH**


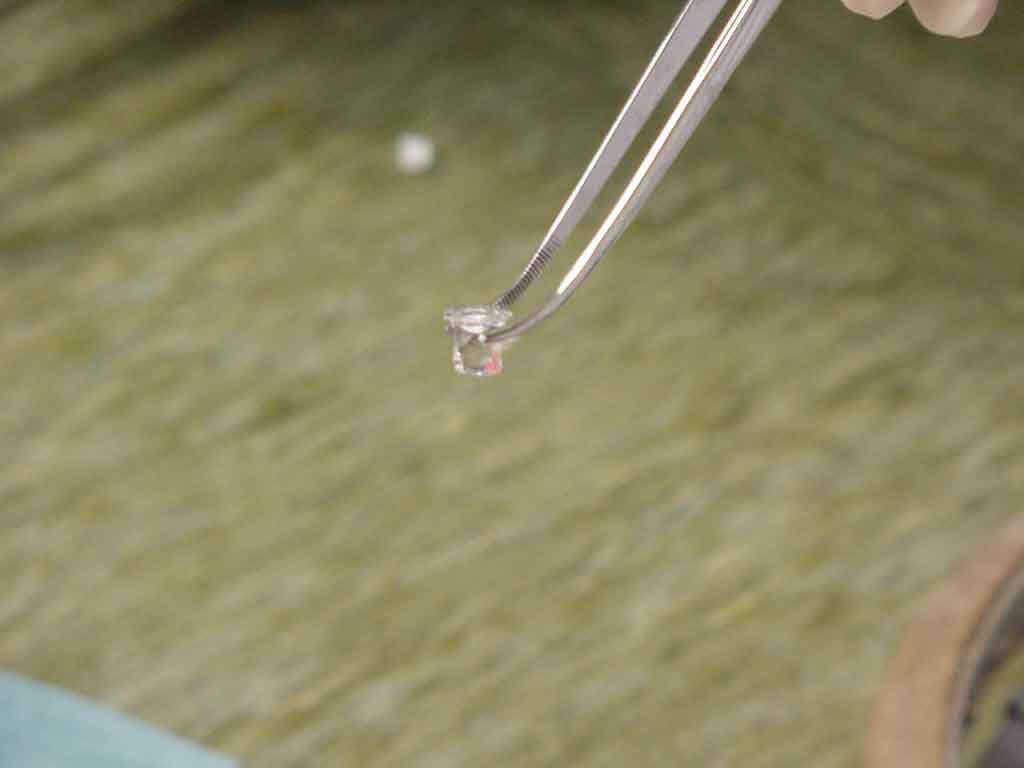


1


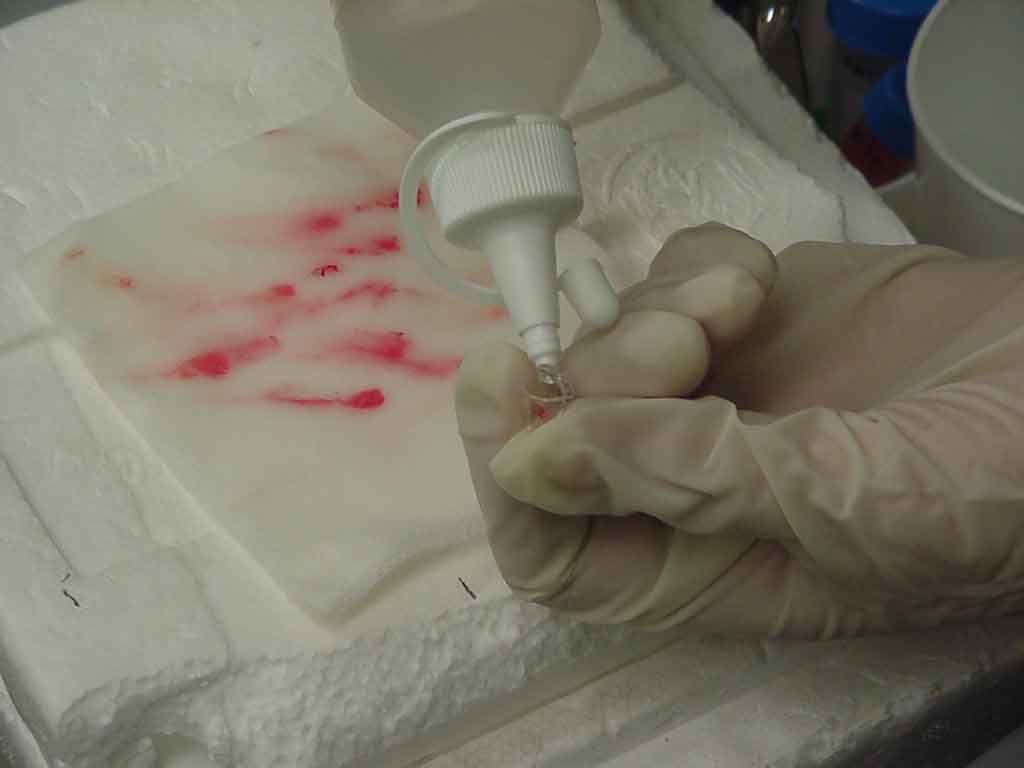


2


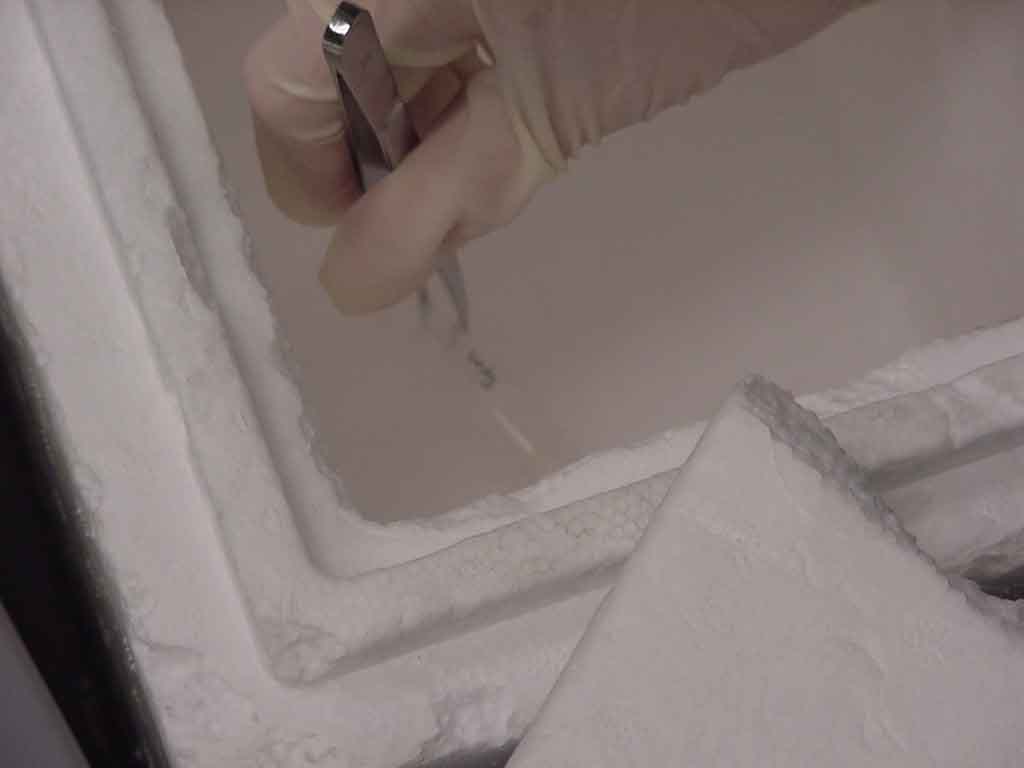


3

- **Step 2.**

Put the synovial tissue samples for IHC (6x) together en bloc on the bottom of two moulds (1).

Fill the moulds completely with Tissue-Tek OCT after the synovial tissue samples have been grouped on the bottom of the moulds (2). It is critical that there is physical contact between different biopsy samples so that it can be analysed as one mega-section. Put the moulds in the labeled cryotubes and let the tissue-tek with the samples for about 2 minutes for complete embedding of the tissue at RT. Snap freeze the cryotubes + moulds in liquid nitrogen (3). Keep the cryotubes in liquid nitrogen for 1 minute, until the Tissue-tek medium in the moulds become white. Leave the cryotubes in liquid nitrogen until storage.

# Step 3.

Put the mould in a labeled cryotube and let the tissue-tek with the samples for about 2 minutes for complete embedding of the tissue at RT. Snap freeze the cryotube + mould in liquid nitrogen. Keep the cryotube in liquid nitrogen for 1 minute, until the Tissue-tek medium in the mould becomes white. Leave the cryotube in liquid nitrogen until storage.

- **Step 4.**


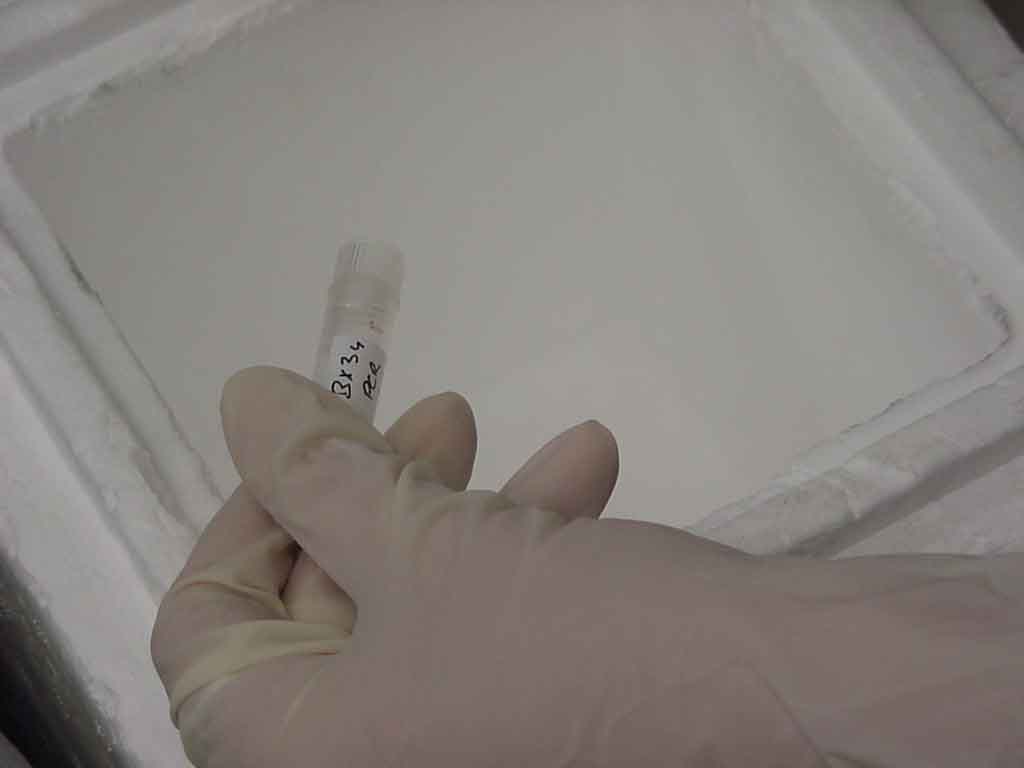

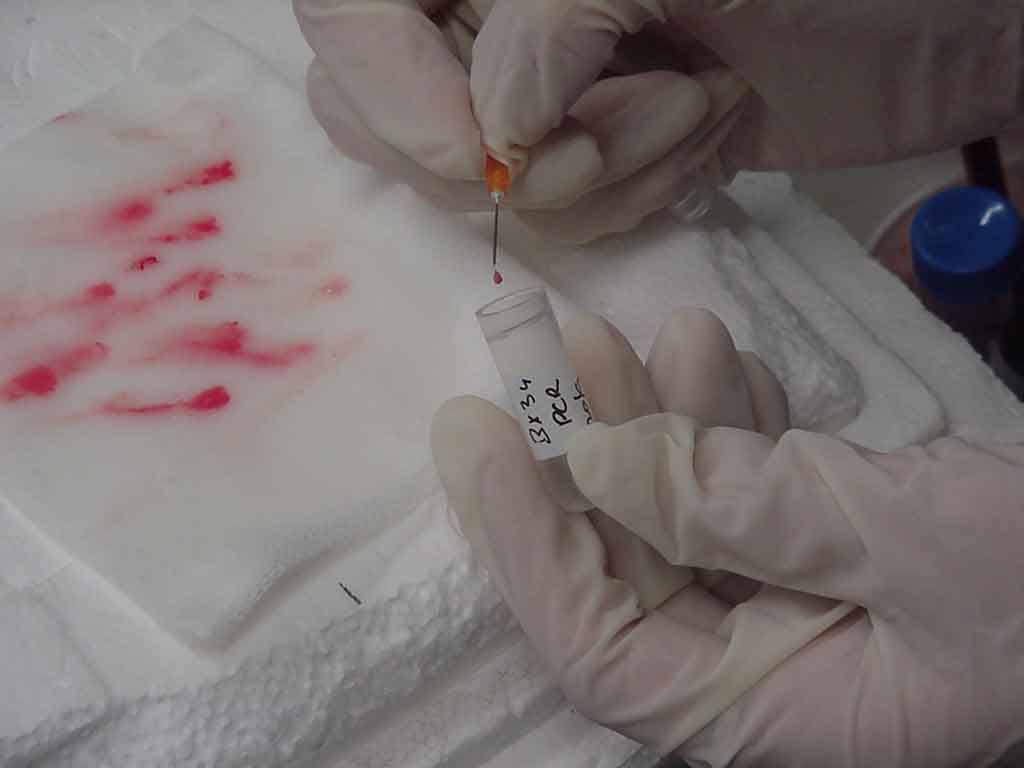
Put the samples for PCR, MA and EMSA directly in a labeled cryotube and freeze immediately in liquid nitrogen.

- **Step 5.**

Put the samples for routine histology (PA) directly in a tube filled with 3.6% formalin for fixation.

All samples should be stored in a liquid nitrogen container. Location of the samples is noted in the Remedy and Excel database.

**APPENDIX 5**

# PSORIASIS AREA AND SEVERITY INDEX (PASI)

| Erythema  Induration  Scaling | None | Mild | Moderate | Severe | Very severe |  |  |
| --- | --- | --- | --- | --- | --- | --- | --- |
| AREA % | 0 | 1-9 | 10-29 | 30-49 | 50-69 | 70-89 | 90-100 |
| SCORE | 0 | 1 | 2 | 3 | 4 | 5 | 6 |

**Erythema (E)**

0 = none

1 = light red

2 = red, but not deep red

3 = very red

4 = extremely red

**Induration (I) (elevation, thickness, infiltration)**

0 = none

1 = slight, but definite to touch

2 = easily palpable with rounded or sloped edges

3 = definitely elevated with hard sharp borders

**Scaling (S)**

0 = none

1 = mainly fine scale, some lesions partially covered

2 = coarser thin scale, most lesions partially covered

3 = coarser thick scale, nearly all lesions covered, rough

4 = very thick scale, all lesions covered, very rough

| **PASI** | Head | Upper limbs | Trunk | Lower limbs |  |
| --- | --- | --- | --- | --- | --- |
| Area % |  |  |  |  |  |
| Erythema |  |  |  |  |  |
| Induration |  |  |  |  |  |
| Scaling |  |  |  |  |  |
| Sum (E+I+S) |  |  |  |  |  |
| Area(Score) |  |  |  |  |  |
| SumxArea |  |  |  |  |  |
| x | x0.1 | x0.2 | x0.3 | x0.4 |  |
| Total | + | + | + | + | = |

| Score of the biopsy site |  |
| --- | --- |
| Erythema | + |
| Induration | + |
| Scaling | + |
| Total | = |

Initials of the investigator: Date:

Reference List

1. Alenius GM, Stenberg B, Stenlund H, Lundblad M, Dahlqvist SR: **Inflammatory joint manifestations are prevalent in psoriasis: prevalence study of joint and axial involvement in psoriatic patients, and evaluation of a psoriatic and arthritic questionnaire.** *J Rheumatol* 2002, **29:** 2577-2582.

2. Gladman DD, Shuckett R, Russell ML, Thorne JC, Schachter RK: **Psoriatic arthritis (PSA)--an analysis of 220 patients.** *Q J Med* 1987, **62:** 127-141.

3. Nash P, Clegg DO: **Psoriatic arthritis therapy: NSAIDs and traditional DMARDs.** *Ann Rheum Dis* 2005, **64 Suppl 2:** ii74-ii77.

4. Fraser A, Fearon U, Billinghurst RC, Ionescu M, Reece R, Barwick T *et al*.: **Turnover of type II collagen and aggrecan in cartilage matrix at the onset of inflammatory arthritis in humans: relationship to mediators of systemic and local inflammation.** *Arthritis Rheum* 2003, **48:** 3085-3095.

5. Ritchlin C, Haas-Smith SA, Hicks D, Cappuccio J, Osterland CK, Looney RJ: **Patterns of cytokine production in psoriatic synovium.** *J Rheumatol* 1998, **25:** 1544-1552.

6. Kupper TS: **Immunologic targets in psoriasis.** *N Engl J Med* 2003, **349:** 1987-1990.

7. Mease PJ: **Psoriatic arthritis therapy advances.** *Curr Opin Rheumatol* 2005, **17:** 426-432.

8. Tak PP, Taylor PC, Breedveld FC, Smeets TJM, Daha MR, Kluin PM *et al*.: **Decrease in cellularity and expression of adhesion molecules by anti-tumor necrosis factor alpha treatment in patients with rheumatoid arthritis.** *Arthritis Rheum* 1996, **39:** 1077-1081.

9. Smeets TJ, Kraan MC, van Loon ME, Tak PP: **Tumor necrosis factor alpha blockade reduces the synovial cell infiltrate early after initiation of treatment, but apparently not by induction of apoptosis in synovial tissue.** *Arthritis Rheum* 2003, **48:** 2155-2162.

10. Goedkoop AY, Kraan MC, Picavet DI, De Rie MA, Teunissen MB, Bos JD *et al*.: **Deactivation of endothelium and reduction in angiogenesis in psoriatic skin and synovium by low dose infliximab therapy in combination with stable methotrexate therapy: a prospective single-centre study.** *Arthritis Res Ther* 2004, **6:** R326-R334.

11. Goedkoop AY, Kraan MC, Teunissen MB, Picavet DI, De Rie MA, Bos JD *et al*.: **Early effects of tumour necrosis factor alpha blockade on skin and synovial tissue in patients with active psoriasis and psoriatic arthritis.** *Ann Rheum Dis* 2004, **63:** 769-773.

12. Gerlag D, Tak PP: **Synovial biopsy.** *BEST PRACT RES CLIN RHEUMATOL* 2005, **19:** 387-400.

13. De Groot J, Te Koppele JM, Harris ED, Jr., Tak PP: **Biological markers.** In *Kelley's Textbook of Rheumatology*. Edited by Harris ED, Jr., Budd RC, Firestein GS, Genovese MC, Sergent JS, Ruddy S *et al*. Philadelphia: Elsevier Saunders; 2005:728-738.

14. Gerlag DM, Haringman JJ, Smeets TJ, Zwinderman AH, Kraan MC, Laud PJ *et al*.: **Effects of oral prednisolone on biomarkers in synovial tissue and clinical improvement in rheumatoid arthritis.** *Arthritis Rheum* 2004, **50:** 3783-3791.

15. Haringman JJ, Gerlag DM, Zwinderman AH, Smeets TJ, Kraan MC, Baeten D *et al*.: **Synovial tissue macrophages: a sensitive biomarker for response to treatment in patients with rheumatoid arthritis.** *Ann Rheum Dis* 2005, **64:** 834-838.

16. Haringman JJ, Vinkenoog M, Gerlag DM, Smeets TJM, Tak PP: **Reliability of computerized image anlaysis for the evaluation of serial synovial biopsies in randomized controlled trials in rheumatoid arthritis.** *Arthritis Res Ther* 2005, **7:** R862-R867.

17. Goedkoop AY, De Rie MA, Picavet DI, Kraan MC, Dinant HJ, Van Kuijk AW *et al*.: **Alefacept therapy reduces the effector T-cell population in lesional psoriatic epidermis.** *Arch Dermatol Res* 2004, **295:** 465-473.

18. Kraan MC, Van Kuijk AW, Dinant HJ, Goedkoop AY, Smeets TJ, De Rie MA *et al*.: **Alefacept treatment in psoriatic arthritis: Reduction of the effector T cell population in peripheral blood and synovial tissue is associated with improvement of clinical signs of arthritis.** *Arthritis Rheum* 2002, **46:** 2776-2784.

19. Tak PP: **Lessons learnt from the synovial tissue response to antirheumatic treatment.** *Rheumatology (Oxford)* 2000, **39:** 817-820.

20. Smeets TJM, Kraan MC, Versendaal H, Breedveld FC, Tak PP: **Analysis of serial biopsies in rheumatoid arthritis patients: description of a control group without clinical improvement after treatment with recombinant human IL-10 or placebo.** *J Rheumatol* 1999, **26:** 2089-2093.

21. Van Holten J, Pavelka K, Vencovsky J, Stahl H, Rozman B, Genovese M *et al*.: **A multicentre, randomised, double blind, placebo controlled phase II study of subcutaneous interferon beta-1a in the treatment of patients with active rheumatoid arthritis.** *Ann Rheum Dis* 2005, **64:** 64-69.

22. Tak PP, Van der Lubbe PA, Cauli A, Daha MR, Smeets TJM, Kluin PM *et al*.: **Reduction of synovial inflammation after anti-CD4 monoclonal antibody treatment in early rheumatoid arthritis.** *Arthritis Rheum* 1995, **38:** 1457-1465.

23. Cunnane G, Madigan A, Murphy E, FitzGerald O, Bresnihan B: **The effects of treatment with interleukin-1 receptor antagonist on the inflamed synovial membrane in rheumatoid arthritis.** *Rheumatology (Oxford)* 2001, **40:** 62-69.
