# Supplementary material for: Soluble Biomarkers of Cartilage and Bone Metabolism in Early Proof of Concept Trials in Psoriatic Arthritis: Effects of Adalimumab Versus Placebo
Source: PLoS One. 2010 Sep 3;5(9):e12556. doi: 10.1371/journal.pone.0012556 (PMC2937309; doi:10.1371/journal.pone.0012556)
Supplement: Table S1 — Demographic and clinical features of the 24 patients with psoriatic arthritis (PsA) enrolled in the study. All values are presented as mean (range) except where indicated otherwise. PA, polyarticular; OA, oligoarticular; DIP, predominant distal interphalangeal; RF, rheumatoid factor; ACPA, anti-citrillunated protein antibody; MTX, methotrexate; ESR, erythrocyte sedimentation rate; CRP, C-reactive protein; DAS28, disease activity score in 28 joints; VAS, visual analogue scale; PASI, psoriasis area end severity index. (0.04 MB RTF) [file pone.0012556.s005.doc]

|  | **Adalimumab (n=12)** | **Placebo**  **(n=12)** |
| --- | --- | --- |
| **Age, years** | 42.8 (21–61) | 47.2 (25–78) |
| **No. men/women** | 9 / 3 | 6 / 6 |
| **PsA disease duration, years** | 5.5 (0.4–14.1) | 8.4 (1.9–18.2) |
| **PsA subtype: PA / OA / DIP, no** | 6 / 6 / 0 | 10 / 1 / 1 |
| **No. (%) RF positive** | 2 (17) | 1 (8) |
| **No. (%) ACPA positive** | 1 (8) | 0 |
| **No. (%) erosive** | 7 (58) | 5 (42) |
| **No. (%) currently receiving MTX** | 7 (58) | 5 (42) |
| **Dose MTX, mg/week** | 18.2 (10–25) | 19.0 (15–25) |
| **ESR, mm/h** | 24.2 (4–66) | 22.4 (3–66) |
| **CRP, mg/litre** | 19.9 (2.3–81.6) | 9.9 (1.3–26.7) |
| **66 swollen joint count** | 6.8 (2-15) | 10.7 (4-19) |
| **DAS28 score** | 4.67 (3.0–5.78) | 5.07 (2.21–6.83) |
| **Patient global assessment of disease activity (VAS 0–100)** | 73 (45–94) | 62.8 (18–92) |
| **Patient assessment of pain (VAS 0–100)** | 72.8 (55–91) | 67.4 (11–89) |
| **PASI** | 5.89 (0–13.8) | 4.72 (0–7.1) |
