# Supplementary material for: Soluble Biomarkers of Cartilage and Bone Metabolism in Early Proof of Concept Trials in Psoriatic Arthritis: Effects of Adalimumab Versus Placebo
Source: PLoS One. 2010 Sep 3;5(9):e12556. doi: 10.1371/journal.pone.0012556 (PMC2937309; doi:10.1371/journal.pone.0012556)
Supplement: Table S2 — Results of markers of collagen type I and collagen type II in placebo and adalimumab treated groups. All values are presented as median (standard deviation, SD). ** P<0.005. NTx, N-terminal telopeptide of type I collagen; PINP, pro-collagen type I N-terminal propeptide; ICTP, C-terminal telopeptide of type I collagen; OC, osteocalcine; MMP-3, matrix metalloproteinase-3; MIA, melanoma inhibitory activity; CPII, C-propeptide of type II collagen; COMP, cartilage oligomeric matrix protein; C2C, Col2-3/4C. (0.04 MB DOC) [file pone.0012556.s006.doc]

|  | **placebo** | | | **adalimumab** | | |
| --- | --- | --- | --- | --- | --- | --- |
| **marker** | **Week 0** | **Week 4** | **Week 12** | **Week 0** | **Week 4** | **Week12** |
| *type I collagen (bone)* |  |  |  |  |  |  |
| **NTx (nM BCE)** | 76.9 (30.0) | 74.9 (29.3) | 66.3 (35.8) | 91.9 (34.3) | 75.3 (23.8) | 92.5 (27.4) |
| **PINP (µg/l)** | 42.6 (34.9) | 47.4 (34.8) | 58.1 (36.6) | 48.1 (15.3) | 52.6 (28.0) | 50.5 (28.1) |
| **ICTP (µg/l)** | 5.10 (2.56) | 5.23 (3.00) | 5.86 (2.83) | 5.19 (1.73) | 4.79 (2.26) | 4.86 (1.89) |
| **OC (ng/ml)** | 15.0 (16.2) | 12.5 (13.3) | 15.0 (14.1) | 17.7 (5.8) | 19.2 (8.0) | 18.4 (8.8) |
| *type II collagen (cartilage)* |  |  |  |  |  |  |
| **MMP-3 (ng/ml)** | 23.9 (33.6) | 23.0 (25.9) | 16.0 (19.2)** | 41.0 (35.1) | 14.5 (12.6)** | 16.3 (11.5)** |
| **MIA (ng/ml)** | 6.25 (2.76) | 6.30 (1.75) | 6.12 (1.74) | 5.77 (3.29) | 6.74 (4.31)** | 6.88 (3.06) |
| **CPII (ng/ml)** | 783 (227) | 772 (280) | 683 (287) | 668 (169) | 765 (167) | 701 (140) |
| **COMP (U/l)** | 12.7 (4.9) | 13.7 (3.7) | 14.8 (3.6) | 12.8 (3.7) | 11.5 (5.3) | 15.5 (2.7) |
| **C2C (ng/ml)** | 163 (29.6) | 163 (32.6) | 157 (37.4) | 160 (22.5) | 162 (29.7) | 186 (28.7) |
