# Supplementary material for: Soluble Biomarkers of Cartilage and Bone Metabolism in Early Proof of Concept Trials in Psoriatic Arthritis: Effects of Adalimumab Versus Placebo
Source: PLoS One. 2010 Sep 3;5(9):e12556. doi: 10.1371/journal.pone.0012556 (PMC2937309; doi:10.1371/journal.pone.0012556)
Supplement: Table S3 — P-values of the repeated measure ANCOVA for each marker, including ESR and CRP. The correlation between each marker, including ESR and CRP, and change in the disease activity evaluated in 28 joints (DAS28) are presented as Spearman rho (P-value). (0.03 MB DOC) [file pone.0012556.s007.doc]

|  | **ANCOVA** | **Correlation** |
| --- | --- | --- |
| **marker** | P-value | with change in DAS28 |
| *inflammation* |  |  |
| **ESR** | 0.001 | 0.737 (P<0.001) |
| **CRP** | 0.01 | 0.755 (P<0.001) |
| *type I collagen (bone)* |  |  |
| **NTx** | 0.082 | 0.422 (P<0.05) |
| **PINP** | 0.16 | -0.097 (n.s.) |
| **ICTP** | 0.5 | 0.303 (n.s.) |
| **OC** | 0.86 | -0.137 (n.s.) |
| *type II collagen (cartilage)* |  |  |
| **MMP-3** | 0.006 | 0.709 (P<0.01) |
| **MIA** | 0.013 | -0.507 (P<0.01) |
| **CPII** | 0.095 | -0.176 (n.s.) |
| **COMP** | 0.4 | 0.345 (n.s.) |
| **C2C** | 0.5 | -0.176 (n.s.) |
